# Low levels of 1,5-anhydro-D-glucitol are associated with vascular endothelial dysfunction in type 2 diabetes

**DOI:** 10.1186/1475-2840-13-99

**Published:** 2014-06-13

**Authors:** Keiichi Torimoto, Yosuke Okada, Hiroko Mori, Yoshiya Tanaka

**Affiliations:** 1 First Department of Internal Medicine, School of Medicine, University of Occupational and Environmental Health, 1-1 Iseigaoka, Yahatanishi-ku, Kitakyushyu-shi 807-8555, Japan

**Keywords:** Reactive hyperemia index (RHI), Type 2 daibetes mellitus, Endothelium, 1,5-anhydro-D-glucitol (1,5-AG)

## Abstract

**Background:**

Vascular endothelial dysfunction is involved in macrovascular disease progression in type 2 diabetes mellitus (T2DM). We reported previously that blood glucose fluctuations, as evaluated by continuous glucose monitoring (CGM), correlate with vascular endothelial function, serving as a marker of vascular endothelial function. However, the use of CGM is limited, suggesting the need for another marker of vascular endothelial function. Here, we investigated the relationship between vascular endothelial dysfunction and blood levels of 1,5-anhydro-D-glucitol (1,5-AG), a marker of both postprandial hyperglycemia and fluctuations in blood glucose.

**Methods:**

In 32 inpatients with T2DM and HbA1c less than 8.0%, the reactive hyperemia index (RHI), an index of vascular endothelial function, was determined by peripheral arterial tonometry. The relationships between RHI and 1,5-AG, blood glucose, lipid metabolism markers, and blood pressure, were examined.

**Results:**

There was a strong correlation between 1,5-AG and natural logarithmic-scaled RHI (L_RHI) (r = 0.55; P = 0.001). However, there was no correlation between L_RHI and HbA1c, fasting blood glucose, IRI, LDL-C, HDL-C, TG, systolic blood pressure, or diastolic blood pressure. Multivariate analysis identified blood 1,5-AG levels to be the only significant and independent determinant of L_RHI.

**Conclusions:**

In T2DM with HbA1c <8.0%, low 1,5-AG levels were associated with vascular endothelial dysfunction, suggesting it is a potentially useful marker for vascular endothelial dysfunction.

**Trial registration:**

UMIN000015317

## Background

Atherosclerosis causes vascular endothelial dysfunction even at an early stage [1], and is known to play a major role in the development and progression of macrovascular disease in type 2 diabetes mellitus (T2DM) [2]. A study using the blood glucose clamp technique showed that endothelial dysfunction progresses via increased oxidative stress associated with fluctuations in blood glucose level [3]. Studies from our laboratory have also reported that the standard deviation (SD) and the mean amplitude of glycemic excursions (MAGE), i.e., indices of fluctuations in blood glucose as determined by continuous glucose monitoring (CGM), correlate significantly with vascular endothelial function [4]. It has become apparent that evaluation of blood glucose fluctuations by CGM is useful for predicting vascular endothelial function. However, the number of patients who use CGM is limited. We also reported previously that hemoglobin A1c (HbA1c) cannot predict vascular endothelial function [4]. Accordingly, we explored the utility of other markers of glycemic control that are evaluated in daily clinical practice as predictors of vascular endothelial function.

The level of 1,5-anhydro-D-glucitol (1,5-AG) in peripheral blood is considered a useful marker of glycemic control [5,6] and a useful predictor of cardiovascular events [7]. The present study was designed to determine the relationship between vascular endothelial function and blood levels of 1,5-AG in patients with T2DM.

## Methods

The present cross-sectional study included 32 patients with T2DM admitted to the University of Occupational and Environmental Health Hospital and Wakamatsu Hospital of the University of Occupational and Environmental Health between January 2012 and November 2013. All patients satisfied the following inclusion criteria: (1) age 20 years and above; (2) HbA1c <8.0%; (3) serum creatinine (Cre) <2.0 mg/dl; and (4) the absence of arrhythmias. Patients with diabetic ketoacidosis, nonketotic hyperosmolar coma, infection, or acute coronary syndrome were excluded from the study. The study protocol was approved by the Human Ethics Review Committee and a signed consent form was obtained from each subject.

### Study design

This study was a cross-sectional study. On the second or third hospital day, fasting blood plasma glucose (FPG) was measured, together with HbA1c, 1,5-AG, immunoreactive insulin (IRI), low-density lipoprotein cholesterol (LDL-C), high-density lipoprotein cholesterol (HDL-C), and triglyceride (TG). Vascular endothelial function was assessed noninvasively using peripheral arterial tonometry (PAT) apparatus (EndoPAT2000, Itamar Medical, Caesarea, Israel) [8]. The reactive hyperemia index (RHI), which is a vasodilatory response to the release of avascularization and reflects the ability to produce nitric oxide (NO) *in vivo* stimulated by vascular shear stress, was measured automatically by PAT [9]. Evaluation of vascular endothelial function with EndoPAT includes the use of the contralateral arm as the control side, and RHI is calculated automatically. This evaluation technique is reported to involve less examiner-dependent variation and to be superior in terms of objectivity compared to the FMD evaluation [10]. No changes were made in oral glucose-lowering agents, lipid metabolism-improving drugs, or antihypertensive drugs from 12 weeks before admission until the end of the study.

The primary endpoint of the study was the relationship between RHI and 1,5-AG, while the secondary endpoints of the study were the relationships between RHI and HbA1c, IRI, homeostasis model of assessment insulin resistance (HOMA-IR), lipid metabolism, and blood pressure.

### Noninvasive vascular function test

The method used for endothelial function measurement using PAT has been described in detail previously [11]. Briefly, after an overnight fast, the subject rested in a temperature- and light-controlled room for a period of 30 min. Baseline pulse amplitude was recorded during a period of 5 min prior to the induction of ischemia. The latter was induced by placing the blood pressure cuff on the upper arm. The opposite arm served as a control. The PAT probes were placed on index finger of each hand. After 5 min, the blood pressure cuff was inflated to 200 mmHg or 60 mmHg above the systolic pressure if systolic pressure was over 140 mmHg for 5 min and then deflated to induce reactive hyperemia. As a measure of reactive hyperemia, RHI was calculated as the ratio of the average amplitude of the PAT signal over 1 min beginning 1.5 min after cuff deflation (control arm, A; occluded arm, C) divided by the average amplitude of the PAT signal over the 2.5-min time period before cuff inflation (baseline) (control arm, B; occluded arm, D). Thus, RHI = (C/D)/(A/B) x baseline correction. Because RHI has a heteroscedastic error structure, we used a natural logarithm transformation in all analyses.

### Measurement of serum lipids, blood HbA1c, 1,5-AG and plasma glucose

Serum lipids were measured using a Hitachi 7350 autoanalyzer (Hitachi Co., Tokyo, Japan). LDL-C, HDL-C, and TG were determined by the enzymatic method, and LDL-C was determined by the direct method. HOMA-IR was calculated using the following formula: HOMA-IR = [fasting IRI (μU/l) × fasting blood glucose (mg/dl)/405]. HbA1c (%) was measured by HPLC using Tosoh HLC-723 G8 (Tosoh Co., Kyoto, Japan), and expressed as National Glycohemoglobin Standardization Program (NGSP) values by adding 0.4% to HbA1c values expressed as the conventional Japanese standard substance (JDS) values [12]. The 1,5-AG level was measured by a colorimetric method (Nippon Kayaku, Tokyo, Japan) using a Bio Majesty JCA-BM 8060 (JEOL, Tokyo, Japan).

### Statistical analyses

All values were expressed as mean ± SD. The Kolmogorov-Smirnov normality test demonstrated that natural logarithmic-scaled RHI (L_RHI), 1,5-AG, HbA1c, FPG, LDL-C, TG, systolic blood pressure (SBP), and diastolic blood pressure (DBP) were normally distributed, whereas IRI and HOMA-IR showed skewed distribution. For intergroup comparisons, the unpaired t-test was used for normally distributed data, the Mann–Whitney U test for data with skewed distributions. To assess potential correlations with L_RHI, the Pearson correlation coefficient was used for data with normal distribution pattern, whereas the Spearman rank-correlation coefficient was used for data with a non-normal distribution. Multivariate analysis was carried out employing the step-up procedure, using L_RHI as the dependent variable, and age, sex, body mass index (BMI), disease duration, use of presence/absence of treatment with α-glucosidase inhibitor treatment or insulin treatment, use of antihypertensive drugs, use of antihyperlipidemic drugs, history of cardiovascular disease (CVD), LDL-C, HDL-C, TG, SBP, DBP, HbA1c, 1,5-AG, and FPG as independent variables. The level of significance was set at *p* < 0.05. SPSS Statistical Software 21.0 (SPSS Inc., Chicago, IL) was used for all statistical analyses.

## Results

### Clinical characteristics

Table 1 lists the clinical features of the 32 (17 men and 15 women) participating patients. The mean L_RHI value was 0.6 ± 0.2 (men: 0.6, women: 0.7) (range: 0.3-1.0), and the mean baseline pulse amplitude was 8.5 ± 4.0 (men: 8.7, women: 8.3), with neither value showing any significant difference between the sexes.

**Table 1:** Patient characteristics

	
Age (years)	64.0 ± 11.1 [37–79]
Gender (male/female)	17/15
Body mass index (kg/m^2^)	26.0 ± 5.3
Duration of diabetes (years)	11.3 ± 12.8 [1–45]
Diabetes therapy	
Diet only	15 (47.0)
Sulfonylurea	9 (28.0)
Pioglitazone	5 (16.0)
Metformin	6 (19.0)
α-glucosidase inhibitor	3 (9.0)
Dipeptidyl peptidase-4 inhibitor	7 (22.0)
Insulin	5 (15.6)
Other treatments	
Lipid-lowering drugs	13 (41.0)
Antihypertensive drugs	14 (44.0)
Current smokers	8 (25.0)
Prevalence cardiovascular disease	7 (22.0)
Systolic blood pressure (mmHg)	135.1 ± 20.0
Diastolic blood pressure (mmHg)	78.3 ± 12.5
LDL-cholesterol (mg/dl)	121.3 ± 23.8
HDL-cholesterol (mg/dl)	50.5 ± 16.9
Triglyceride (mg/dl)	119.8 ± 60.1
HbA1c (%)	7.3 ± 0.5 [6.2-7.9]
1,5-anhydro-D-glucitol (μg/ml)	7.4 ± 3.5 [2.6-15.3]
Fasting plasma glucose (mg/dl)	128.1 ± 25.3 [75–185]
Immunoreactive insulin (μU/ml)^a^	8.4 ± 8.6
HOMA-IR^a^	2.6 ± 2.4
L_RHI	0.6 ± 0.2

Data are mean ± SD [range], n, or (%). ^a^n = 27.

LDL, low-density lipoprotein; HDL, high-density lipoprotein; HbA1c, hemoglobin A1c; HOMA-IR, homeostasis model assessment as an index of insulin resistance; L_RHI, the natural logarithmic scaled reactive hyperemia index.

### Relationship between L_RHI and markers of diabetes control and nonglycemic metabolic variables

The relationship between L_RHI and clinical markers of glycemic control is shown in Table 2. Univariate analysis showed a statistically significant relationship between L_RHI and 1,5-AG (r = 0.55; P = 0.001). On the other hand, L_RHI did not correlate with HbA1c, FPG, IRI, or HOMA-IR. L_RHI also did not correlate with LDL-C, HDL-C, TG, SBP, or DBP.

**Table 2:** Correlation coefficients between L_RHI and clinical markers of glycemia and various nonglycemic metabolic variables

	**HbA1c**	**1,5-AG**	**FPG**	**IRI**	**HOMA-IR**	**LDL-C**	**HDL-C**	**TG**	**SBP**	**DBP**
1,5-AG (n = 32)	−0.38*									
FPG (n = 32)	0.18	−0.24								
IRI (n = 27)	0.26	0.19	−0.15							
HOMA-IR (n = 27)	0.27	0.17	−0.02	0.98**						
LDL-C (n = 32)	−0.02	−0.28	−0.17	−0.03	−0.04					
HDL-C (n = 32)	−0.14	−0.06	0.07	−0.43*	−0.45*	−0.40*				
TG (n = 32)	−0.01	−0.04	−0.17	0.27	0.20	0.28	−0.70**			
SBP (n = 32)	−0.11	−0.05	−0.39*	0.11	0.06	0.34	−0.08	−0.01		
DBP (n = 32)	−0.09	−0.13	−0.14	0.32	0.27	0.18	−0.06	−0.01	0.72**	
L_RHI (n = 32)	−0.08	0.55**	−0.25	0.14	0.06	0.08	−0.04	0.01	0.03	−0.19

Data are results of Pearson correlation analysis for normally distributed variables and Spearman rank correlation for variables with skewed distribution.

*P < 0.05, **P < 0.01.

Abbreviations as in Table 1. 1,5-AG, 1,5-anhydro-D-glucitol; FPG, Fasting plasma glucose; IRI, Immunoreactive insulin; HDL-C, high-density lipoprotein cholesterol; TG, triglyceride; SBP, systolic blood pressure; DBP, diastolic blood pressure.

In the next step, we compared the relationship between L_RHI and treatment with and without sulfonylurea, pioglitazone, metformin, α-glucosidase inhibitor, dipeptidyl peptidase-4 inhibitor, and insulin. There was no relationship between L_RHI and treatment with and without sulfonylurea (0.64 ± 0.09 vs 0.61 ± 0.04, p = 0.818), pioglitazone (0.71 ± 0.13 vs 0.60 ± 0.04, p = 0.311), metformin (0.77 ± 0.09 vs 0.58 ± 0.04, p = 0.060), and dipeptidyl peptidase-4 inhibitor treatment (0.72 ± 0.11 vs 0.59 ± 0.04, p = 0.210). On the other hand, L_RHI was significantly higher in the α-glucosidase inhibitor-treated group (0.86 ± 0.04, n = 3) than -untreated group (0.59 ± 0.04, n = 29) (P = 0.042), and significantly lower in the insulin-treated group (0.38 ± 0.04, n = 5) than -untreated group (0.66 ± 0.04, n = 27) (P = 0.006).

Multivariate analysis, using L_RHI as the dependent variable and age, sex, BMI, disease duration, use of presence/absence of treatment with α-glucosidase inhibitor treatment or insulin treatment, use of antihypertensive drugs, use of antihyperlipidemic drugs, history of CVD, LDL-C, HDL-C, TG, SBP, DBP, HbA1c, 1,5-AG, and FPG as the independent variables, identified 1,5-AG as the only significant and independent determinant of L_RHI (adjusted multiple R^2^ = 0.277, standardization coefficient β = 0.548, t = 3.149, P = 0.001) (Table 3).

**Table 3:** Linear multivariate analyses with L_RHI as the dependent variables

**Variables**	**Unstandardized coefficients**		**Standardized coefficients β**	**t**	**P value**	**95% CI**
	**B**	**SE**				
Intercept	0.370	0.077		4.819	<0.001	0.213-0.527
1,5-AG	0.034	0.009	0.548	3.149	0.001	0.015-0.053
Adjusted Multiple R^2^	0.277					

Multivariate stepwise regression analysis with L_RHI as the dependent variable and age, gender, BMI, duration of the disease, use of presence/absence of treatment with α-glucosidase inhibitor treatment or insulin treatment, antihypertensive drugs, lipid-lowering drugs, prevalence CVD, LDL-cholesterol, HDL-cholesterol, triglyceride, systolic blood pressure, HbA1c, 1,5-AG, and fasting plasma glucose as the independent variables.

Abbreviations as in Tables 1 and 2: SE, Standard error; CVD, cardiovascular disease; 95% CI, 95% confidence interval.

Because the incidence of cardiovascular events is reportedly increased significantly in patients with L_RHI of ≤0.4 [13], the subjects were divided into two groups; the low L_RHI group (L_RHI ≤0.4, n = 7) and the high L_RHI group (L_RHI >0.4, n = 25). The 1,5-AG level was statistically significantly lower in the low L_RHI group (4.7 ± 1.8 μg/ml) compared with high L_RHI group (8.1 ± 3.6 μg/ml) (p = 0.023). However, HbA1c was not statistically significantly different between the two groups (low group:7.3 ± 0.3%, high group:7.2 ± 0.5%, p = 0.855).

## Discussion

The main finding of the present study was that vascular endothelial function parameter L_RHI correlates significantly with 1,5-AG in T2DM patients with HbA1c <8.0%.

In fact, 1,5-AG blood level correlate with postprandial hyperglycemia in patients with HbA1c <8.0%, in both type 1 diabetes mellitus [14] and T2DM [15]. In addition, the use of CGM has demonstrated a significant correlation with MAGE and indices of postprandial hyperglycmia in patients with HbA1c <8.0% [16]. 1,5-AG has been clinically used as a marker of changes in blood glucose level. Sakamoto et al. [17] used this parameter to compare changes in blood glucose level according to glucose-lowering therapy, similar to CGM data. Furthermore, since low 1,5-AG levels are associated with coronary artery disease, 1,5-AG has also been used to identify patients at high risk of cardiovascular disease [18]. Furthermore, blood 1,5-AG levels correlated inversely with serum Cre levels especially in patients with Cre ≥2.0 mg/dl [15]. Based on the reported relationship between 1,5-AG blood levels and HbA1c <8.0% and Cre <2.0 mg/dl described above [14,15], we focused in this study on patients with HbA1c <8.0%.

Blood 1,5-AG level is a marker of glycemic control and accurately reflects rises and falls in urinary glucose excretion. When 1,5-AG leaks into the urine along with excessive excretion of glucose, the result is a decreased concentration in the blood. In other words, blood glucose levels correlate inversely with those of 1,5-AG [19]. Because glucose is immediately excreted into the urine even after a very short period of postprandial hyperglycemia, 1,5-AG was previously reported to serve as a marker of glycemic control that reflects fluctuations in blood glucose [20-22]. On the other hand, in relation to complications, 1,5-AG reportedly correlates with albuminuria [23] and the cardio-ankle vascular index [24], independent of HbA1c. A cohort study that followed subjects for a mean of 11 years demonstrated 1,5-AG to be a strong predictor of CVD [7]. Emoto et al. [25] reported that improvement in flow mediated dilation during a period of α-glucosidase administration was associated with 1,5-AG improvement in patients with type 2 diabetes accompanied by coronary artery disease. However, there is only limited information on 1,5-AG and vascular endothelial function, and no study free of the effects of drug treatments has yet been reported. In the present study, vascular endothelial function, evaluated by PAT, correlated strongly with 1,5-AG, suggesting the 1,5-AG level is a potentially useful predictor of vascular endothelial function, as well as a marker of fluctuations in blood glucose, in patients with HbA1c <8%. Based on this result, we believe that 1,5-AG should be evaluated in patients with HbA1c <8%, and that treatment of postprandial hyperglycemia and multidisciplinary risk management for atherosclerosis should be provided to patients with low 1,5-AG levels.

L_RHI is mainly an index of vascular endothelial function that reflects vasodilatory responses to NO, an endothelium-dependent vasodilatory factor [26]. Vascular endothelial function determined by L_RHI is reported to allow detection of atherosclerosis at an early stage and to correlate with coronary atherosclerosis [11], as well as to predict coronary artery disease [13]. As for the relationship between glucose metabolism and vascular endothelial function, it is known that increased oxidative stress and fluctuations in blood glucose together worsen vascular endothelial dysfunction [3], and indices of fluctuations in blood glucose, such as MAGE and postprandial hyperglycemia, correlate strongly with vascular endothelial function [4]. Specifically, fluctuations in blood glucose and postprandial hyperglycemia are considered to be major factors favoring the progression of vascular endothelial dysfunction in glucose metabolism. On the other hand, it was recently reported that 1,5-AG correlates with MAGE and postprandial hyperglycemia in CGM [16,27]. The findings of this study indicate that 1,5-AG correlates strongly with vascular endothelial function because it is a marker of glycemic control that reflects, particularly fluctuations in blood glucose and postprandial hyperglycemia.

This study has several limitations. First, this study did not include control subjects free of diabetes mellitus. Second, we did not evaluate the relationship between vascular endothelial function with either oxidative stress or inflammation. Third, about half of the subjects were on treatment intended to improve lipid metabolism. Although a relationship between L_RHI and blood pressure or the ratio of TC to HDL-C was found in a large-scale cohort study [28], no relationship was found between L_RHI and blood pressure or lipid metabolism in our study, presumably because most of our patients were on antihypertensive drugs or statins. Fourth, the determination coefficient of the independent variable was low for the model employed in this study, with the adjusted R^2^ being equal to 0.277 in multivariate analysis. The sample size was relatively small, and therefore the obtained results require further confirmation in a larger number of patients.

## Conclusions

The present study demonstrated that low levels of 1,5-AG, a marker of fluctuations in blood glucose level, are associated with vascular endothelial dysfunction in patients with HbA1c <8.0%.

## Abbreviations

1,5-AG: 1,5-anhydro-D-glucitol; CGM: Continuous glucose monitoring; CVD: Cardiovascular disease; DBP: Diastolic blood pressure; FPG: Fasting plasma glucose; HbA1c: Hemoglobin A1c; HDL: High-density lipoprotein; HOMA-IR: Homeostasis model of assessment insulin resistance; IRI: Immunoreactive insulin; JDS: Japanese standard substance; L_RHI: Natural logarithmic-scaled reactive hyperemia index; LDL: Low-density lipoprotein; MAGE: Mean amplitude of glycemic excursions; PAT: Peripheral vascular arterial tonometry; RHI: Reactive hyperemia index; SBP: Systolic blood pressure; T2DM: Type 2 diabetes mellitus; TG: Triglycerides.

## Competing interests

The authors declare no conflict of interest.

## Authors’ contributions

All authors listed on the manuscript participated in the design of the study and in writing the manuscript. KT performed the statistical analysis. All authors read and approved the final manuscript.

## References

[B1] Ross R Atherosclerosis-An inflammatory disease N Engl J Med 1999 340 115 126 10.1056/NEJM199901143400207 9887164

[B2] Xu J Zou MH Molecular insights and therapeutic targets for diabetic endothelial dysfunction Circulation 2009 120 1266 1286 10.1161/CIRCULATIONAHA.108.835223 19786641 PMC2910587

[B3] Ceriello A Esposito K Piconi L Ihnat MA Thorpe JE Testa R Boemi M Giugliano D Oscillating glucose is more deleterious to endothelial function and oxidative stress than mean glucose in normal and type 2 diabetic patients Diabetes 2008 57 1349 1354 10.2337/db08-0063 18299315

[B4] Torimoto K Okada Y Mori H Tanaka Y Relationship between fluctuations in glucose levels measured by continuous glucose monitoring and vascular endothelial dysfunction in type 2 diabetes mellitus Cardiovasc Diabetol 2013 12 1 10.1186/1475-2840-12-1 23280391 PMC3557219

[B5] Yamanouchi T Tachibana Y Akanuma H Minoda S Shinohara T Moromizato H Miyashita H Akaoka I Origin and disposal of 1,5-anhydroglucitol, a major polyol in the human body Am J Physiol 1992 263 E268 E273 1514606 10.1152/ajpendo.1992.263.2.E268

[B6] Yamanouchi T Akanuma Y Serum 1,5-anhydroglucitol (1,5 AG): new clinical marker for glycemic control Diabetes Res Clin Pract 1994 24 S261 S268 7859616 10.1016/0168-8227(94)90259-3

[B7] Watanabe M Kokubo Y Higashiyama A Ono Y Miyamoto Y Okamura T Serum 1,5-anhydro-D-glucitol levels predict first-ever cardiovascular disease: an 11-year population-based cohort study in Japan, the Suita study Atherosclerosis 2011 216 477 483 10.1016/j.atherosclerosis.2011.02.033 21414624

[B8] Kuvin JT Patel AR Sliney KA Pandian NG Sheffy J Schnall RP Karas RH Udelson JE Assessment of peripheral vascular endothelial function with finger arterial pulse wave amplitude Am Heart J 2003 146 168 174 10.1016/S0002-8703(03)00094-2 12851627

[B9] Celermajer DS Reliable endothelial function testing: at our fingertips? Circulation 2008 117 2428 2430 10.1161/CIRCULATIONAHA.108.775155 18474821

[B10] Flammer AJ1 Anderson T Celermajer DS Creager MA Deanfield J Ganz P Hamburg NM Lüscher TF The assessment of endothelial function: from research into clinical practice Circulation 2012 126 753 767 10.1161/CIRCULATIONAHA.112.093245 22869857 PMC3427943

[B11] Bonetti PO Pumper GM Higano ST Holmes DR Jr Kuvin JT Lerman A Noninvasive identification of patients with early coronary atherosclerosis by assessment of digital reactive hyperemia J Am Coll Cardiol 2004 44 2137 2141 10.1016/j.jacc.2004.08.062 15582310

[B12] The Committee of Japan Diabetes Society on the diagnostic criteria of diabetes mellitus Report of the Committee on the classification and diagnostic criteria of diabetes mellitus J Diabetes Investig 2010 1 212 228 24843435 10.1111/j.2040-1124.2010.00074.x PMC4020724

[B13] Rubinshtein R Kuvin JT Soffler M Lennon RJ Lavi S Nelson RE Pumper GM Lerman LO Lerman A Assessment of endothelial function by noninvasive peripheral arterial tonometry predicts late cardiovascular adverse events Eur Heart J 2010 31 1142 1148 10.1093/eurheartj/ehq010 20181680

[B14] Mehta SN Schwartz N Wood JR Svoren BM Laffel LM Evaluation of 1,5-anhydroglucitol, hemoglobin A1c, and glucose levels in youth and young adults with type 1 diabetes and healthy controls Pediatr Diabetes 2012 13 278 284 10.1111/j.1399-5448.2011.00830.x 22060802 PMC3767297

[B15] Dungan KM 1,5-anhydroglucitol (GlycoMark) as a marker of short-term glycemic control and glycemic excursions Expert Rev Mol Diagn 2008 8 9 19 10.1586/14737159.8.1.9 18088226

[B16] Sun J Dou JT Wang XL Yang GQ Lü ZH Zheng H Ma FL Lu JM Mu YM Correlation between 1,5-anhydroglucitol and glycemic excursions in type 2 diabetic patients Chin Med J (Engl) 2011 124 3641 3645 22340217

[B17] Sakamoto M1 Nishimura R Irako T Tsujino D Ando K Utsunomiya K Comparison of vildagliptin twice daily vs. sitagliptin once daily using continuous glucose monitoring (CGM): crossover pilot study (J-VICTORIA study) Cardiovasc Diabetol 2012 11 92 10.1186/1475-2840-11-92 22867630 PMC3471040

[B18] Fujiwara T1 Yoshida M Yamada H Tsukui T Nakamura T Sakakura K Wada H Arao K Lower 1,5-anhydroglucitol is associated with denovo coronary artery disease in patients at high cardiovascular risk Heart Vessels 2014 Apr 2 [Epub ahead of print] 10.1007/s00380-014-0502-y 24691699

[B19] Yamanouchi T Akaoka I Akanuma Y Akanuma H Miyashita E Mechanism for acute reduction of 1,5-anhydroglucitol in rats treated with diabetogenic agents Am J Physiol 1990 258 E423 E427 2138419 10.1152/ajpendo.1990.258.3.E423

[B20] Yamanouchi T Moromizato H Shinohara T Minoda S Miyashita H Akaoka I Estimation of plasma glucose fluctuation with a combination test of hemoglobin A1c and 1,5-anhydroglucitol Metabolism 1992 41 862 867 10.1016/0026-0495(92)90168-A 1640864

[B21] Yamanouchi T Ogata N Tagaya T Kawasaki T Sekino N Funato H Akaoka L Miyashita H Clinical usefulness of serum 1,5-anhydroglucitol in monitoring glycaemic control Lancet 1996 347 1514 1518 10.1016/S0140-6736(96)90672-8 8684103

[B22] Kishimoto M Yamasaki Y Kubota M Arai K Morishima T Kawamori R Kamada T 1,5-Anhydro-D-glucitol evaluates daily glycemic excursions in well-controlled NIDDM Diabetes Care 1995 18 1156 1159 10.2337/diacare.18.8.1156 7587851

[B23] Yamanouchi T Kawasaki T Yoshimura T Inoue T Koshibu E Ogata N Funato H Akaoka I Miyashita H Relationship between serum 1,5-anhydroglucitol and urinary excretion of N-acetylglucosaminidase and albumin determined at onset of NIDDM with 3-year follow-up Diabetes Care 1998 21 619 624 10.2337/diacare.21.4.619 9571353

[B24] Ohira M Endo K Oyama T Yamaguchi T Ban N Kawana H Nagayama D Nagumo A Saiki A Murano T Watanabe H Miyashita Y Shirai K Improvement of postprandial hyperglycemia and arterial stiffness upon switching from premixed human insulin 30/70 to biphasic insulin aspart 30/70 Metabolism 2011 60 78 85 10.1016/j.metabol.2010.06.001 20667560

[B25] Emoto T Sawada T Hashimoto M Kageyama H Terashita D Mizoguchi T Mizuguchi T Motodi Y Iwasaki M Taira K Okamoto H Matsuo Y Kim SK Takarada A Yokoyama M Effect of 3-month repeated administration of miglitol on vascular endothelial function in patients with diabetes mellitus and coronary artery disease Am J Cardiol 2012 109 42 46 10.1016/j.amjcard.2011.08.005 21944671

[B26] Nohria A Gerhard-Herman M Creager MA Hurley S Mitra D Ganz P Role of nitric oxide in the regulation of digital pulse volume amplitude in humans J Appl Physiol 2006 101 545 548 10.1152/japplphysiol.01285.2005 16614356

[B27] Dungan KM Buse JB Largay J Kelly MM Button EA Kato S Wittlin S 1,5-anhydroglucitol and postprandial hyperglycemia as measured by continuous glucose monitoring system in moderately controlled patients with diabetes Diabetes Care 2006 29 1214 1219 10.2337/dc06-1910 16731998

[B28] Hamburg NM Keyes MJ Larson MG Vasan RS Schnabel R Pryde MM Mitchell GF Sheffy J Vita JA Benjamin EJ Cross-sectional relations of digital vascular function to cardiovascular risk factors in the Framingham Heart Study Circulation 2008 117 2467 2474 10.1161/CIRCULATIONAHA.107.748574 18458169 PMC2734141

